# Multilevel Lumbar CT Attenuation Beyond L1: Comparison with QCT-Derived Volumetric Bone Mineral Density and Prevalent Fragility Fracture Status in a Diagnostic Referral Cohort

**DOI:** 10.3390/diagnostics16162640

**Published:** 2026-08-19

**Authors:** Julian Ramin Andresen, Stephan Heisinger, Thomas Haider, Hans-Christof Schober, Reimer Andresen

**Affiliations:** 1 Department of Orthopaedics, University Clinic for Orthopaedics and Trauma Surgery, Medical University of Vienna, Waehringer Guertel 18-20, 1090 Vienna, Austria; 2 Department of Trauma Surgery, University Clinic for Orthopaedics and Trauma Surgery, Medical University of Vienna, Waehringer Guertel 18-20, 1090 Vienna, Austria; 3 Practice for Orthopedics and Osteology, OrthoCoast, Hufelandstrasse 1, 17438 Wolgast, Germany; 4 Institute for Diagnostic and Interventional Radiology/Neuroradiology, Westkuestenklinikum Heide, Academic Teaching Hospital of the Universities of Kiel, Luebeck and Hamburg, Esmarchstrasse 50, 25746 Heide, Germany

**Keywords:** osteoporosis, Hounsfield units, quantitative computed tomography, vertebral fracture, trabecular bone, multilevel assessment, bone mineral density, diagnostic referral cohort

## Abstract

**Background/Objectives:** CT-based trabecular attenuation in Hounsfield units (HU) is used as a surrogate marker of bone quality, but most approaches rely on a single vertebral level, usually L1, where local abnormalities may limit reliability and availability. This study evaluated whether multilevel lumbar, reference-adjusted HU assessment beyond isolated L1 improves the identification of QCT-defined osteoporosis and the discrimination of prevalent fragility fracture status. **Methods:** Between 2021 and March 2024, 800 patients referred for evaluation of bone mineral density underwent QCT of the lumbar spine with a calibration phantom. Cancellous attenuation was measured in HU at L1–L3 using manually positioned ellipsoid regions of interest, with predefined adjacent levels (T12, L4) substituted when a target vertebra was unsuitable. Single-level HU, the mean multilevel HU value and the lowest valid HU value were compared with QCT-defined osteoporosis and prevalent fragility fracture status (vertebral or sacral) using ROC analyses and pairwise DeLong testing. **Results:** Mean multilevel HU correlated closely with mean vBMD (Spearman *ρ* = 0.988; *p* < 0.001); as both derive from the same acquisition, the same vertebral bodies and the same phantom, this reflects a strong association between two related measurements rather than validation against an independent standard. Osteoporosis was present in 483 patients (60.4%). Mean multilevel HU was associated with osteoporosis with an AUC of 0.996 (95% CI, 0.993–0.998) at an internally derived cut-off of ≤99.6 HU. In the 699 patients with a valid L1 measurement, in whom a paired comparison was possible, mean multilevel HU exceeded isolated L1-HU (0.992; *p* = 0.0028). For fracture status, the AUC was 0.975 (0.966–0.983) at ≤80.5 HU, without advantage over L1-HU (*p* = 0.816). A valid value was obtainable in all 800 patients versus 699 (87.4%) with L1 alone. All patients with a fracture had a vBMD below the osteoporosis threshold, so this endpoint is structurally dependent on the densitometric one. **Conclusions:** Multilevel lumbar HU assessment provides a practical marker of trabecular bone quality beyond isolated L1. Its principal advantage is measurement availability rather than a clinically meaningful gain in discrimination. The reported thresholds are internally derived, cohort- and protocol-specific, and require external validation.

## 1. Introduction

Osteoporosis is a systemic skeletal disorder characterized by reduced bone mass, deterioration of trabecular microarchitecture, and increased susceptibility to fragility fractures [1]. Worldwide, osteoporosis is estimated to affect approximately 200 million women, and one in three women and one in five men over the age of 50 are expected to sustain an osteoporosis-related fracture [2]. Within the axial skeleton, vertebral fractures represent one of the most frequent and clinically relevant manifestations of osteoporosis and are estimated to occur approximately every 22 s worldwide in adults over 50 years of age [2,3]. They often occur after minimal or no trauma, remain underdiagnosed in routine clinical care, and are associated with pain, spinal deformity, functional decline, impaired quality of life, and increased morbidity [3,4,5,6]. Importantly, the occurrence of one vertebral fracture substantially increases the risk of subsequent vertebral fractures, thereby marking a critical point in the osteoporotic fracture cascade [7,8]. Established clinical risk factors for insufficiency and fragility fractures include advanced age and postmenopausal status, prior fragility fracture, vitamin D deficiency with secondary hyperparathyroidism or osteomalacia, chronic inflammatory or renal bone disease, ankylosing spondylitis, long-term corticosteroid exposure, and previous pelvic radiotherapy [2,7,8,9,10,11,12,13,14,15]. In elderly inpatient cohorts, osteoporosis-related risk parameters also include female sex, advanced age, impaired nutritional status, reduced physical function, and lower serum 25-hydroxyvitamin D levels [16]. Bone mineral density assessment remains central to osteoporosis diagnosis and fracture risk stratification. Dual-energy X-ray absorptiometry (DXA) is widely used in routine care; however, lumbar DXA can be influenced by degenerative changes, vascular calcifications, osteophytes, and vertebral deformities. Quantitative computed tomography (QCT) allows volumetric assessment of trabecular bone mineral density and is less affected by cortical and extraskeletal degenerative changes [17,18]. Established QCT thresholds classify trabecular lumbar spine vBMD values above 120 mg/cm^3^ as normal, values between 80 and 120 mg/cm^3^ as osteopenic, and values below 80 mg/cm^3^ as osteoporotic [18,19]. Despite its diagnostic value, QCT is not universally available and is usually not performed as a screening tool in patients who have already undergone routine CT imaging for other indications.

Opportunistic CT-based bone assessment has therefore gained increasing relevance. Routine CT examinations contain quantitative attenuation information that can be extracted from cancellous bone in Hounsfield units (HU) without additional radiation exposure, patient time, or imaging costs. Previous biomechanical and clinical studies have demonstrated correlations between vertebral HU values, bone mineral density, vertebral strength, and osteoporotic fracture status [20,21,22,23,24,25,26,27,28,29]. Ullrich et al. also reported very high inter-rater reliability of HU measurements and close correlation with QCT-derived bone mineral density [30]. Large-scale opportunistic screening studies further support the use of CT attenuation as a practical surrogate marker of bone quality, particularly at the lumbar spine [22,26,29]. Current clinical positions also acknowledge the value of opportunistic CT attenuation measurements, with L1 values below 100 HU indicating a high likelihood of osteoporosis and values above 150 HU suggesting normal bone density [31].

However, most available approaches rely on a single vertebral level, usually L1. This is practical and easy to implement, but it may be vulnerable to local confounders such as vertebral deformity, previous fracture, focal sclerosis, hemangioma, degenerative endplate changes, vascular structures, cement augmentation, or metal artifacts. In addition, lumbar trabecular bone density is not necessarily homogeneous across vertebral levels. A multilevel approach using L1-L3 may therefore provide a more robust estimate of regional cancellous bone quality than a single-level measurement, particularly in elderly patients with degenerative spine disease or prevalent vertebral fractures [32,33,34,35,36].

Recent work from our group has shown that opportunistic HU measurements of the lumbar spine and proximal femur can discriminate patients with sacral insufficiency fractures and can identify osteoporotic bone density ranges with clinically meaningful thresholds [37]. Other studies from the same research line demonstrated correlations between lumbar HU measurements, QCT-derived bone mineral density, interobserver reliability, and fracture distribution within the axial skeleton [33,34,35,36]. This question is also clinically relevant because recent publications continue to highlight variability in HU thresholds and region-of-interest placement for CT-based bone assessment [38,39]. Nevertheless, it remains unclear whether a multilevel lumbar HU approach provides additional diagnostic value over isolated L1 measurement when directly compared with QCT-derived vBMD and prevalent fragility fracture status in a large clinical cohort.

The aim of the present study was therefore to evaluate CT-based trabecular attenuation measurements at L1, L2, and L3 in comparison with QCT-derived volumetric bone mineral density and prevalent fragility fracture status in a diagnostic referral cohort. Specifically, we investigated whether single-level HU measurements, the mean multilevel HU value, or the lowest valid lumbar/adjacent HU value best identify QCT-defined osteoporosis and discriminate prevalent fragility fracture status. We hypothesized that multilevel HU assessment provides a simple, widely applicable method for CT-based bone assessment beyond isolated L1 measurement, primarily by increasing the proportion of patients in whom a valid measurement can be obtained and by reducing the influence of local vertebral confounders.

## 2. Materials and Methods

### 2.1. Study Design and Ethics Approval

This single-center retrospective study was approved by the Ethics Committee of the Medical Faculty of Christian-Albrechts-University of Kiel (reference number D 471/24; date of approval 28 March 2024). The study was conducted in accordance with the principles of the Declaration of Helsinki and applicable institutional requirements for retrospective imaging analyses. Reporting follows the STROBE recommendations for observational studies.

### 2.2. Patient Population

Between 2021 and March 2024, patients referred for dedicated evaluation of bone mineral density underwent quantitative CT of the lumbar spine with a calibration phantom. All examinations and all image analyses were performed at a single center, the Institute for Diagnostic and Interventional Radiology/Neuroradiology, Westkuestenklinikum Heide, Germany. The data were subsequently processed in a multicenter setting across the authors’ institutions; with respect to patient recruitment, imaging and image analysis the study is therefore single-center. The uniform referral indication was the assessment of whether osteoporosis and, where applicable, prevalent osteoporotic fractures were present, prior to planning further therapeutic measures. Patients were eligible if they were 18 years of age or older and had a technically adequate phantom-calibrated examination permitting trabecular measurement of at least two vertebral bodies between T12 and L4. Patients were excluded if they had a known malignant disease, a pre-existing pronounced scoliosis, or a history of spinal surgery. All consecutive patients meeting these criteria during the study period were included; no further selection was applied. No patient was excluded on account of previous spinal surgery or pronounced scoliosis, as none was encountered; vertebral body deformities in this cohort were the consequence of osteoporotic compression fractures. A total of 800 patients referred for evaluation of osteoporosis were included.

Patients were referred from outpatient units for bariatric surgery, gynecology, geriatrics, neurosurgery, orthopedics, and trauma surgery.

All patients were referred for dedicated assessment of bone mineral density and were examined with a calibration phantom in place, and all shared a single referral indication. The cohort therefore represents a diagnostic referral population with a high pre-test probability of osteoporosis and does not correspond to a general screening population undergoing CT for an unrelated clinical indication. The multilevel measurement concept evaluated here is transferable to the opportunistic setting, but the diagnostic estimates and thresholds reported in this study apply to the population and protocol studied. Baseline characteristics of the cohort, overall and stratified by sex and by prevalent fragility fracture status, are given in Table 1.

### 2.3. Imaging and Measurements

All examinations were acquired on a GE Revolution EVO 64-slice CT scanner (GE HealthCare, Chicago, IL, USA) using the dedicated QCT acquisition protocol specified by the software manufacturer (Mindways Software, Austin, TX, USA). Acquisition was performed at 120 kVp with a slice thickness and slice spacing of 2.0 mm and a helical pitch closest to 1. Tube current was controlled by automatic dose modulation (Auto mA) with a noise index of 20 HU. Scan time was 2 s or less, the scan field of view was 50 cm, and the display field of view was 38 cm, centered at X = 0 and Y = 0, with a 512 × 512 display matrix. Images were reconstructed with the Standard abdominal kernel; a bone kernel was not used. Sagittal reformats of the same slice thickness were generated from this acquisition and were used for the trabecular attenuation measurements, so that axial and reformatted images had identical section thickness. A lateral localizer covering T10 to L5/S1 was obtained to prescribe the axial series. No intravenous contrast medium was administered in any examination; all acquisitions were unenhanced. The calibration phantom was positioned in a cutout pad directly beneath the patient with a bolus bag interposed, and all examinations were acquired at a standardized QCT table height. Before patient examinations, the scanner was characterized using the quality assurance phantom, and quality assurance scans were required to return a “Pass” result.

**Figure 1:**
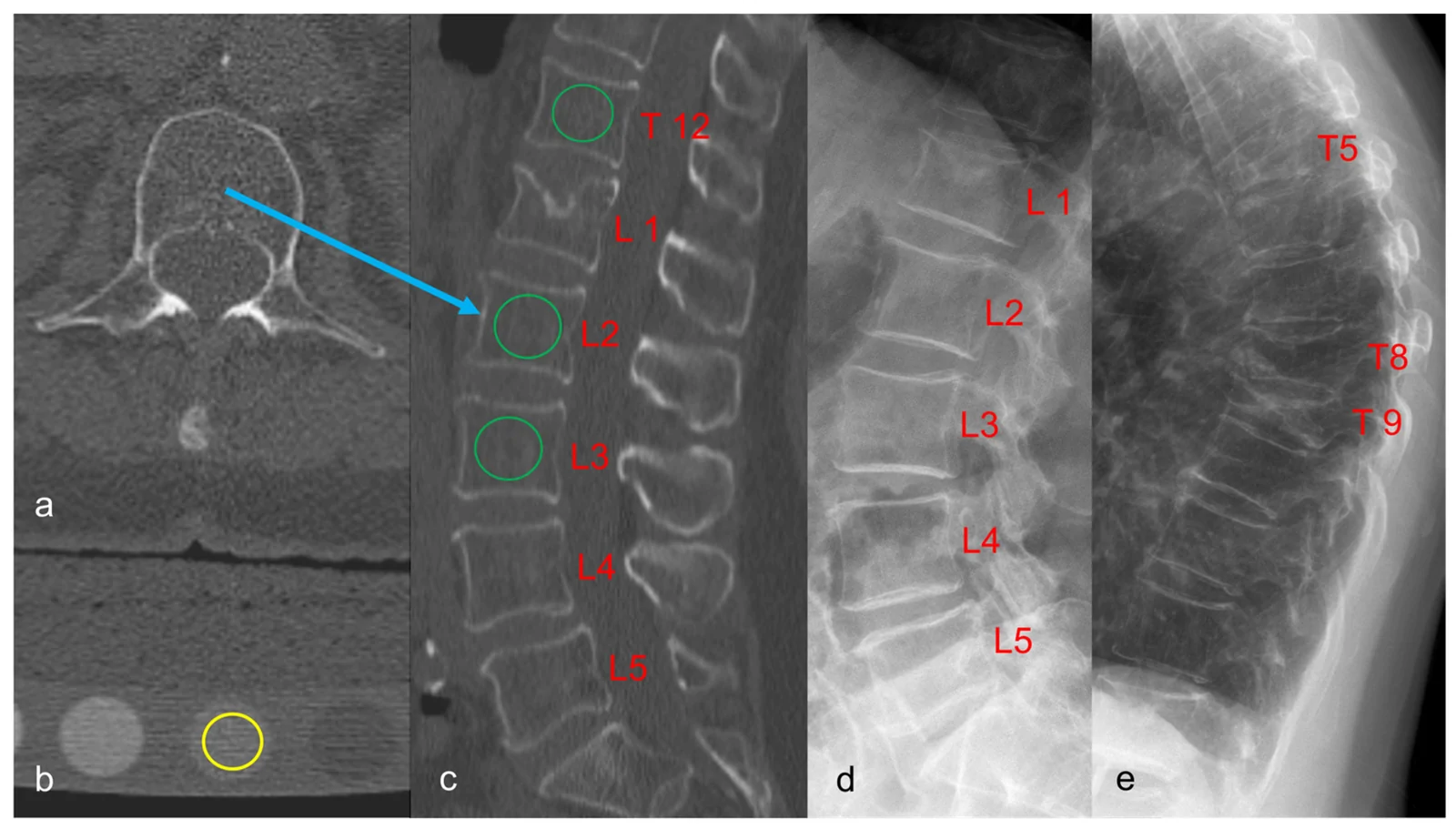
Representative imaging example in a 74-year-old woman with clinically manifest osteoporosis. (**a**) Axial mid-vertebral CT image at L2 demonstrating cancellous trabecular bone; the blue arrow marks the L2 level, corresponding to the middle measurement ROI on the sagittal reformat in (**c**). (**b**) Reference phantom used for calibration, with an ROI (yellow circle) placed in the central density insert. The measured attenuation was 51 HU, consistent with the manufacturer-specified target value of 50 HU (SD approximately 4 HU). (**c**) Sagittal reformatted CT image showing the circular/ellipsoid ROIs (green circles) used for HU measurement at T12, L2, and L3; T12 was used instead of L1 because of L1 endplate collapse. The mean attenuation was 56 HU, within the osteoporotic range. Lateral lumbar (**d**) and thoracic (**e**) spine radiographs demonstrate chronic compression fractures at L1, T9, T8 and T5.

Trabecular volumetric bone mineral density (vBMD, mg/cm^3^) was determined by QCT using Mindways 3D Volumetric QCT Spine software—Mindways Software, Austin, TX, USA, which reports bone mineral density as equivalent aqueous K2HPO4 density. Within the software, each vertebral body was rotated interactively in the sagittal, coronal and axial planes until it was aligned as an upright box, after which the automatically generated trabecular volume of interest was inspected and adjusted where necessary. QCT analyses were carried out by trained radiographic technologists and were subsequently reviewed and verified by a radiologist (R.A.). Trabecular attenuation measurements were performed at the lumbar vertebral levels using manually placed cancellous ROIs, as illustrated in Figure 1.

After pseudonymization, additional cancellous bone attenuation measurements were obtained in HU in the same vertebral bodies, resulting in a total of 2400 vertebral body measurements. Measurements were performed using a manually positioned circular/ellipsoid region of interest (ROI) in the midvertebral cancellous compartment on sagittal reformatted CT images, with a slice thickness of 2.0 mm and a window setting of center 400/window 1600. The size of the region of interest was not fixed but was adapted to the size of the individual vertebral body, and was made as large as the trabecular compartment permitted while keeping a margin of approximately 1 mm from the cortical bone of the superior and inferior endplates, so that cortical bone and the basivertebral venous plexus were excluded. Each region of interest was positioned on the sagittal reformatted image, and every region-of-interest position was subsequently verified on axial and coronal reformatted images before the measurement was accepted. A vertebral body was regarded as technically unsuitable and was replaced by a predefined adjacent level if it showed a prevalent fracture, focal sclerosis, a hemangioma or other focal lesion, or advanced degenerative endplate change precluding reliable placement of the region of interest within unaffected cancellous bone. Cement augmentation and metallic implants did not occur in this cohort, because patients with previous spinal surgery were not eligible. Reference-body adjustment was performed by measuring the central density tube of the simultaneously scanned reference phantom on the same CT series; vertebral cancellous attenuation values were corrected against this reference measurement to reduce acquisition- and scanner-related variability. The reported HU values therefore represent reference-adjusted cancellous attenuation values, as illustrated in Figure 1.

Substitution followed a fixed hierarchical rule: where L1 was unsuitable, T12 was measured in its place; where L3 was unsuitable, L4 was measured in its place. L2 had no designated substitute. This range corresponds to the T11–L4 acquisition range specified in the QCT protocol. The frequency with which each substitute level was used is reported in Table 2 and Section 3.1.

**Relationship between the two measurements.** Both vBMD and HU values were derived from the same CT acquisition, from the same vertebral bodies, and with reference to the same calibration phantom. vBMD was computed by the QCT software from an automated three-dimensional trabecular volume of interest using the manufacturer’s phantom calibration equation, whereas HU values were measured separately by a human reader on sagittal reformatted images using manually placed elliptical regions of interest and were corrected against the measured attenuation of the central density insert of the same phantom. The two measurements therefore share the acquisition, the anatomical region and the calibration reference, and differ only in the sampled volume, the reconstruction plane, the operator and the software. They are consequently not statistically independent, The association between them is therefore reported and interpreted as an association between two related measurements, and not as validation against an independent reference standard. No formal agreement analysis, such as Bland–Altman analysis of the differences between the two measurements, was performed, and the correlation and regression statistics reported here do not by themselves establish agreement between the two methods.

**Readers and blinding.** All image data were pseudonymized before attenuation measurement. All trabecular attenuation measurements were performed by a single reader (R.A.), a board-certified radiologist with many years of experience in musculoskeletal diagnostic imaging, working on the pseudonymized data without access to the QCT-derived vBMD values. Vertebral body fractures and vertebral height reductions were graded independently by a second investigator (H.C.S.) with many years of experience in osteology. The two investigators had no access to each other’s results: the reader performing the attenuation measurements was blinded to the radiographic fracture assessment, and the investigator grading the fractures was blinded to the attenuation and vBMD values. Because all attenuation measurements were performed by a single reader and were not repeated, neither inter-rater nor intra-rater agreement could be quantified in the present cohort.

Additional lateral radiographs of the thoracic and lumbar spine were evaluated for detection and grading of vertebral body fractures according to the semiquantitative method of Genant et al. [40]. This classification considers the extent of vertebral height loss: grade 1 indicates mild anterior, middle, or posterior height reduction of 20–25%; grade 2 indicates moderate height reduction of 25–40%; and grade 3 indicates severe height reduction greater than 40% compared with adjacent vertebrae. When a sacral insufficiency fracture was clinically suspected, simultaneously available axial CT images and MRI examinations were additionally evaluated. Prevalent vertebral fractures were also apparent on the CT examinations, but the number of fractures and the severity grade were determined on the radiographs. Radiographic fracture assessment was available for all 800 patients, so fracture ascertainment was complete and uniform across the cohort.

### 2.4. Statistical Analysis

Statistical analyses were performed using IBM SPSS Statistics, Version 23.0 (IBM Corp., Armonk, NY, USA). Specific comparisons of correlated ROC curves were additionally performed using the method described by DeLong et al. [41]. Quantitative variables were reported as mean, standard deviation (SD), and number (*n*) when approximately normally distributed. Nonparametric data were reported as median with first and third quartiles (Q1–Q3). Normality was assessed using the Shapiro–Wilk test. All *p*-values were based on two-sided tests, and *p* < 0.05 was considered statistically significant. The prespecified primary pairwise ROC comparison was the mean multilevel HU parameter versus isolated L1-HU; additional pairwise comparisons were interpreted descriptively.

The central HU parameters were the individual valid vertebral-level measurements at L1, L2, and L3; the arithmetic mean of all valid multilevel measurements, including predefined adjacent-level substitutions (T12 or L4) when a target L1–L3 vertebra was fractured or technically unsuitable; and the lowest valid lumbar/adjacent HU value. The arithmetic mean of all valid measurements is referred to as the mean multilevel HU value, and this single term is used consistently throughout the manuscript, including in the abstract, the tables and the figure legends. Analogously, QCT values were evaluated as individual vertebral measurements and as mean lumbar values. QCT-defined osteoporosis was defined as trabecular vBMD < 80 mg/cm^3^; values of 80–120 mg/cm^3^ were classified as osteopenic and values > 120 mg/cm^3^ as normal [18,19].

**Definition of the fracture endpoint.** The patient-level fracture endpoint was the presence of at least one prevalent osteoporotic fragility fracture of the thoracolumbar spine or the sacrum, and is referred to throughout as prevalent fragility fracture status. Sacral insufficiency fractures were included in this endpoint; six patients had an isolated sacral fracture without a vertebral fracture. A prespecified sensitivity analysis was performed in which the endpoint was restricted to vertebral body fractures and these six patients were classified as fracture-free. The terms vertebral body fracture and sacral insufficiency fracture are used only when referring to individual fracture sites.

Associations between HU values and corresponding QCT values were first assessed using Pearson or Spearman correlation coefficients, depending on data distribution. Because multiple vertebral measurements were available per patient, regression models accounting for within-patient clustering were additionally used. For this purpose, QCT values were modeled using generalized estimating equations with an exchangeable working correlation structure and a robust sandwich variance estimator. In addition, a linear mixed model with patients as random intercepts and vertebral level as a fixed factor was used to evaluate systematic differences between L1, L2, and L3 and intraindividual variability of HU values; the intraclass correlation coefficient was derived from the resulting variance components. The results of both models are reported in Section 3.3.

The diagnostic performance of single-level and multilevel HU parameters was evaluated by ROC analyses for two endpoints: QCT-defined osteoporosis and prevalent fragility fracture status. For L1, L2, and L3, the mean multilevel HU value, and the lowest valid HU value, the area under the curve (AUC) with 95% confidence interval (CI), the optimal Youden-index cut-off, sensitivity, specificity, positive predictive value (PPV), and negative predictive value (NPV) were reported where available. AUC values of individual measurements were compared pairwise with the mean multilevel HU parameter and with L1-HU using the DeLong test for correlated ROC curves [41].

**Confidence intervals and additional analyses.** Confidence intervals for areas under the curve were calculated using the method of DeLong et al. [41]; confidence intervals for sensitivity, specificity and predictive values were calculated using the Wilson score method; and confidence intervals for the Youden-index cut-off values were calculated by percentile bootstrapping with 2000 resamples. Three additional analyses were performed. First, a complete-case analysis was restricted to patients in whom valid measurements were available at all of L1, L2 and L3 for both HU and QCT. Second, diagnostic performance was examined separately in prespecified strata defined by sex, by age below or from 65 years, and by BMI below or from 30 kg/m^2^. Third, the association between mean multilevel HU and prevalent fragility fracture status was examined in a multivariable logistic regression model adjusted for age, sex and BMI, with odds ratios and 95% confidence intervals reported. Stratified and adjusted analyses were exploratory and were not corrected for multiple comparisons.

## 3. Results

### 3.1. Measurement Availability and Use of Adjacent Substitute Levels

A valid trabecular HU measurement was obtained at L1 in 699 of 800 patients (87.4%), at L2 in 738 (92.3%) and at L3 in 750 (93.8%). The mean multilevel HU value and the lowest valid HU value could be calculated in all 800 patients (100%). Adjacent substitute levels were required in 179 patients (22.4%): T12 replaced L1 in 102 patients (12.8%) and L4 replaced L3 in 112 patients (14.0%), with both substitutions required in 35 patients. A complete set of valid L1–L3 measurements was available for HU in 621 patients (77.6%) and for both HU and QCT in 610 patients (76.3%). Substitution was strongly related to fracture status, being required in 137 of 339 patients with a prevalent fragility fracture (40.4%) but in only 42 of 461 patients without fracture (9.1%).

In 787 of 800 patients (98.4%), the HU mean and the QCT mean were derived from an identical set of vertebral levels. In the remaining 13 patients (1.6%), the two means were based on different level combinations, because a vertebra that was technically unsuitable for manual ROI placement was not necessarily excluded by the QCT software, or the reverse. All analyses were repeated after excluding these 13 patients, with unchanged results.

Measurement characteristics, the distribution of bone density and attenuation values, and fracture burden are summarized in Table 2.

**Table 2:** Measurement characteristics, bone density and fracture status of the study population, overall and stratified by sex and by prevalent fragility fracture status. vBMD, volumetric bone mineral density; HU, Hounsfield units. Fracture site counts include vertebral body fractures and sacral fracture sites; a bilateral sacral insufficiency fracture is counted as two sites.

Characteristic	Total (*n* = 800)	Women (*n* = 659)	Men (*n* = 141)	No Fracture (*n* = 461)	Fracture (*n* = 339)
**Measurement characteristics**					
Vertebrae measured per patient, median	3	3	3	3	3
Complete valid L1–L3, *n* (%)	621 (77.6)	507 (76.9)	114 (80.9)	419 (90.9)	202 (59.6)
≥1 adjacent substitute level used, *n* (%)	179 (22.4)	152 (23.1)	27 (19.1)	42 (9.1)	137 (40.4)
—T12 substituted for L1, *n* (%)	102 (12.8)	87 (13.2)	15 (10.6)	24 (5.2)	78 (23.0)
—L4 substituted for L3, *n* (%)	112 (14.0)	97 (14.7)	15 (10.6)	21 (4.6)	91 (26.8)
Valid L1-HU available, *n* (%)	699 (87.4)	573 (86.9)	126 (89.4)	443 (96.1)	256 (75.5)
**Bone density and attenuation**					
Mean vBMD, mg/cm^3^, mean ± SD	79.7 ± 38.3	76.9 ± 37.4	92.7 ± 40.2	103.3 ± 32.5	47.5 ± 15.4
Mean vBMD, mg/cm^3^, median (Q1–Q3)	72.2 (53.8–102.7)	69.4 (52.1–95.4)	92.5 (68.4–127.9)	96.0 (75.0–122.4)	50.2 (34.9–57.9)
Mean vBMD, mg/cm^3^, range	10.3–191.5	10.3–191.5	20.1–170.5	56.5–191.5	10.3–79.3
QCT normal (>120 mg/cm^3^), *n* (%)	131 (16.4)	84 (12.7)	47 (33.3)	131 (28.4)	0 (0)
QCT osteopenic (80–120 mg/cm^3^), *n* (%)	186 (23.2)	153 (23.2)	33 (23.4)	186 (40.3)	0 (0)
QCT osteoporotic (<80 mg/cm^3^), *n* (%)	483 (60.4)	422 (64.0)	61 (43.3)	144 (31.2)	339 (100)
Mean multilevel HU, mean ± SD	96.8 ± 47.0	93.3 ± 45.8	113.2 ± 49.1	126.2 ± 38.7	56.9 ± 19.8
Mean multilevel HU, median (Q1–Q3)	87.0 (64.2–124.7)	83.2 (64.0–117.1)	111.6 (82.3–156.1)	115.7 (95.5–150.4)	59.6 (41.3–71.7)
Mean multilevel HU, range	15.4–243.1	15.4–243.1	23.9–217.5	59.0–243.1	15.4–113.7
**Fracture status**					
≥1 prevalent fragility fracture, *n* (%)	339 (42.4)	298 (45.2)	41 (29.1)	0 (0)	339 (100)
Total fracture sites recorded, *n*	1051	905	146	0	1051

### 3.2. Association Between QCT-vBMD, HU Values, Age and Fracture Burden

Complete data for mean QCT-derived volumetric bone mineral density (vBMD), mean multilevel HU value, and age were available for all 800 included patients. Because the investigated variables were not normally distributed, Spearman rank correlation was used as the primary correlation analysis; Pearson correlation coefficients were calculated as supplementary analyses.

Mean QCT-vBMD and mean multilevel HU showed a very strong positive correlation. The Spearman correlation coefficient was *ρ* = 0.988 with *p* < 0.001. The supplementary Pearson correlation showed an almost identical association, with *r* = 0.989 and *p* < 0.001. The relationship was close to a fixed proportionality (mean multilevel HU = 0.33 + 1.211 × mean vBMD), with a residual standard deviation of 6.9 HU; the individual ratio of HU to vBMD had a mean of 1.214 and a standard deviation of 0.108, corresponding to a coefficient of variation of 8.9%.

Age was negatively correlated with mean QCT-vBMD. The Spearman correlation was *ρ* = −0.676 with *p* < 0.001; the supplementary Pearson correlation was *r* = −0.708 with *p* < 0.001. Age was also negatively correlated with mean multilevel HU, with *ρ* = −0.672 and *r* = −0.705, respectively, both with *p* < 0.001.

Fracture burden showed a strong negative correlation with mean multilevel HU (Spearman *ρ* = −0.842; *p* < 0.001) and with mean QCT-vBMD (*ρ* = −0.839; *p* < 0.001), whereas age was moderately positively correlated with fracture burden (*ρ* = 0.495; *p* < 0.001). These values are based on the corrected fracture counts described in Section 3.5. All correlations are summarized in Table 3.

### 3.3. Vertebral-Level Models Accounting for Within-Patient Clustering

Because up to three vertebral measurements were available per patient, models accounting for within-patient clustering were fitted to the 2176 L1–L3 measurements with paired HU and QCT values from 800 patients. In the generalized estimating equation model with an exchangeable working correlation structure, vBMD increased by 0.772 mg/cm^3^ per Hounsfield unit (standard error 0.006; *p* < 0.001), with an estimated working correlation of 0.813.

In the linear mixed model with patients as random intercepts and vertebral level as a fixed factor, mean attenuation at L1 was 98.6 HU; values were 1.6 HU lower at L2 (standard error 0.3; *p* < 0.001) and 4.2 HU lower at L3 (standard error 0.3; *p* < 0.001), indicating a small but consistent craniocaudal gradient. The corresponding gradient for vBMD was −0.6 mg/cm^3^ at L2 (*p* = 0.001) and −2.0 mg/cm^3^ at L3 (*p* < 0.001). Between-patient variance greatly exceeded within-patient variance for both parameters, with an intraclass correlation coefficient of 0.99 for HU and 0.99 for vBMD. Among the 621 patients with complete L1–L3 measurements, the mean within-patient standard deviation across the three levels was 4.0 HU (median 2.6 HU) and the median absolute difference between L1 and L3 was 3.5 HU.

### 3.4. Diagnostic Performance of Single-Level and Multilevel HU Parameters

The diagnostic performance of individual and multilevel HU parameters was assessed by comparing isolated L1-, L2-, and L3-HU measurements, the mean valid multilevel HU value, and the lowest valid HU value.

QCT-defined osteoporosis, defined as mean trabecular vBMD < 80 mg/cm^3^, was present in 483 of 800 patients (60.4%).

All HU parameters showed very strong positive correlations with mean QCT-vBMD. Spearman correlations were *ρ* = 0.986 for L1, *ρ* = 0.984 for L2, *ρ* = 0.981 for L3, *ρ* = 0.988 for mean multilevel HU, and *ρ* = 0.983 for the lowest HU value. The strongest correlation with QCT-vBMD was therefore observed for mean multilevel HU.

Unless stated otherwise, all estimates reported in this section and in Table 4 refer to the full cohort of 800 patients, each parameter evaluated in the patients in whom it was obtainable. All HU parameters were closely associated with QCT-defined osteoporosis in this cohort. AUC values were 0.992 for L1, 0.993 for L2, 0.996 for L3, 0.996 for mean multilevel HU, and 0.996 for the lowest HU value. Mean multilevel HU reached an internally derived optimal cut-off of ≤99.6 HU (95% CI, 99.1–103.0), with 96.3% sensitivity (95% CI, 94.2–97.6) and 99.1% specificity (95% CI, 97.3–99.7). The lowest HU value had an optimal cut-off of ≤96.1 HU, with 96.9% sensitivity and 97.2% specificity.

In direct pairwise comparison, mean multilevel HU showed a small but statistically significant advantage over isolated L1-HU for QCT-defined osteoporosis (AUC 0.995 versus 0.992; DeLong *p* = 0.0028), and a nominal advantage over L2-HU (*p* = 0.027). No significant difference was observed compared with L3-HU or the lowest HU value. The direct comparison is presented in detail in Section 3.6.

For prevalent fragility fracture status, all HU parameters also showed high diagnostic performance. AUC values were 0.971 for L1, 0.970 for L2, 0.970 for L3, 0.975 for mean multilevel HU, and 0.976 for the lowest valid HU value. Mean multilevel HU reached an optimal cut-off of ≤80.5 HU (95% CI, 77.4–83.2), with 91.4% sensitivity (95% CI, 88.0–94.0) and 93.5% specificity (95% CI, 90.9–95.4). The lowest valid HU value reached an optimal cut-off of ≤75.6 HU, with 89.1% sensitivity and 95.0% specificity. Full diagnostic estimates with confidence intervals are given in Table 4.

In the prespecified sensitivity analysis restricted to vertebral body fractures, in which the six patients with an isolated sacral fracture were classified as fracture-free, the area under the curve for mean multilevel HU was 0.979 (95% CI, 0.972–0.986) at a cut-off of ≤80.5 HU, with 92.2% sensitivity and 92.9% specificity. These fracture estimates must be read together with Section 3.7: every patient with a prevalent fracture had a vBMD below the osteoporosis threshold, so the fracture endpoint is structurally dependent on the densitometric endpoint and the corresponding estimates are not an independent test against a clinical event. These fracture estimates must be read together with Section 3.7: every patient with a prevalent fracture had a vBMD below the osteoporosis threshold, so in this selected diagnostic referral population the fracture endpoint is structurally dependent on the densitometric endpoint. The corresponding estimates therefore do not constitute independent validation against a clinical outcome, and they carry no implication that the cut-off predicts future or incident fractures. The inclusion of sacral insufficiency fractures in the primary endpoint therefore did not materially influence the results.

### 3.5. Fracture Distribution and Fracture Burden

Of the 800 included patients, 461 were fracture-free, corresponding to 57.6% of the cohort. At least one prevalent fragility fracture was present in 339 patients, corresponding to 42.4%. A single fracture site was documented in 71 patients, two fractures in 66 patients, three fractures in 88 patients, four fractures in 37 patients, five fractures in 45 patients, six fractures in 19 patients, and seven fractures in 13 patients (Table 5). Patients with a prevalent fracture were markedly older than those without (73.2 ± 10.7 versus 60.0 ± 12.8 years) and had substantially lower vBMD (47.5 ± 15.4 versus 103.3 ± 32.5 mg/cm^3^) and attenuation (56.9 ± 19.8 versus 126.2 ± 38.7 HU); these characteristics are tabulated in Table 1 and Table 2.

Topographic fracture distribution (Table 6) showed that 51 patients had fractures exclusively in the thoracic spine and 53 patients exclusively in the lumbar spine. In 119 patients, both the thoracic and lumbar spine were affected. Combined involvement of the spine and sacrum was present in 110 patients, including thoracic spine and sacrum in 47 patients, lumbar spine and sacrum in 22 patients, and combined thoracic spine, lumbar spine, and sacral involvement in 41 patients. Isolated sacral fracture was present in 6 patients.

A total of 834 vertebral body fractures were identified based on documented fracture locations. Of these, 470 were located in the thoracic spine and 364 in the lumbar spine. Thus, 56.4% of vertebral body fractures were thoracic and 43.6% were lumbar.

The most frequent fracture locations were L1 with 135 fractures, T7 with 104 fractures, T12 with 98 fractures, T8 with 88 fractures, L2 with 86 fractures, L3 with 72 fractures, and L4 with 60 fractures. Fractures therefore clustered particularly at the thoracolumbar junction and in the mid-thoracic spine.

According to the Genant classification [40], 400 vertebral body fractures were grade 1, corresponding to 48.0% of vertebral fractures. Grade 2 fractures were documented 365 times, corresponding to 43.8%. Grade 3 fractures were documented 69 times, corresponding to 8.3%. Mild and moderate vertebral fractures therefore predominated, whereas severe fractures were less frequent.

**Definition of the fracture counts.** The per-patient fracture counts reported in Table 5 and the vertebral body fracture counts reported in Table 7 enumerate different entities. Table 5 reports all recorded fracture sites, comprising 834 vertebral body fractures and 116 sacral insufficiency fractures, of which 101 were bilateral and are recorded as two sites, giving 1051 expected sites. The diagnostic analyses are unaffected by this distinction, since they use the binary fracture endpoint rather than the fracture count.

### 3.6. Direct Comparison of L1-HU and Mean Multilevel HU

To assess whether isolated L1-HU or the mean multilevel HU value had higher diagnostic performance, both parameters were directly compared. L1-HU was available in 699 of 800 patients, whereas the mean multilevel HU value was available in all 800 patients. Therefore, the direct pairwise comparison was performed in the 699 patients with available L1-HU.

Because a paired comparison requires both parameters in the same patients, this analysis is restricted to the 699 patients with a valid L1 measurement. The areas under the curve reported here therefore differ slightly from those in Table 4, which refer to the full cohort of 800 patients: for prevalent fragility fracture status the value for mean multilevel HU is 0.971 in this restricted set and 0.975 in the full cohort. For QCT-defined osteoporosis, mean multilevel HU showed higher diagnostic performance than isolated L1-HU. The AUC of L1-HU was 0.992, whereas mean multilevel HU reached an AUC of 0.995. The difference between both ROC curves was statistically significant in the DeLong test (*p* = 0.0028). The optimal L1-HU cut-off was ≤102.9 HU, with 96.2% sensitivity and 98.0% specificity. The optimal mean HU cut-off was ≤99.6 HU, with 96.2% sensitivity and 99.0% specificity. In absolute terms, the difference in the area under the curve was 0.003.

Correlation with QCT-derived vBMD was also slightly stronger for mean multilevel HU than for isolated L1-HU. The Spearman correlation between L1-HU and QCT-vBMD was *ρ* = 0.986, whereas the Spearman correlation between mean multilevel HU and QCT-vBMD was *ρ* = 0.988. The corresponding Pearson correlation coefficients were *r* = 0.985 for L1-HU and *r* = 0.989 for mean HU (*n* = 699).

For prevalent fragility fracture status, no significant difference was observed between isolated L1-HU and mean multilevel HU. The AUC was 0.971 for L1-HU and 0.971 for mean HU. The pairwise comparison of ROC curves did not show a statistically significant difference (*p* = 0.816). The optimal cut-off was ≤81.3 HU for L1-HU and ≤80.2 HU for mean HU.

The complete-case analysis restricted to the 610 patients with valid measurements at all of L1, L2 and L3 for both HU and QCT reproduced these findings: the area under the curve for QCT-defined osteoporosis was 0.992 for L1-HU and 0.995 for mean multilevel HU (DeLong *p* = 0.0005), and for prevalent fragility fracture status 0.968 for both parameters (*p* = 0.96). The results are therefore not attributable to the use of adjacent substitute levels. All comparisons are summarized in Table 8.

### 3.7. Stratified and Adjusted Analyses

Diagnostic performance was examined separately within prespecified strata (Table 9). Discrimination was consistently high across all subgroups. The threshold associated with prevalent fragility fracture shifted markedly with age, from ≤93.5 HU in patients under 65 years to ≤80.5 HU in patients aged 65 years and older. In the smaller strata comprising 141 men, 176 patients with a BMI of 30 kg/m^2^ or above and 352 patients under 65 years for the osteoporosis endpoint, the groups were completely separated by mean multilevel HU, so that the area under the curve reached unity and the confidence interval is degenerate; These values of 1.000 result from complete separation in relatively small subgroups and are not evidence of perfect diagnostic accuracy or of generalizability; complete separation is a small-sample phenomenon that would not be expected to persist in an independent cohort.

In the multivariable logistic regression model for prevalent fragility fracture status (Table 10), the association with mean multilevel HU was essentially unchanged after adjustment for age, sex and BMI, whereas the univariable age effect was no longer present after adjustment. The area under the curve of the predicted probabilities from the fully adjusted model was 0.979, compared with 0.975 for mean multilevel HU alone in the same 800 patients; age, sex and BMI therefore add very little once attenuation is known. This value is not to be confused with the area under the curve of 0.979 reported in Section 3.4, which refers to mean multilevel HU alone under the vertebral-fracture-only endpoint definition.

**Relationship between the two endpoints.** All 339 patients with a prevalent fragility fracture had a mean vBMD below the 80 mg/cm^3^ osteoporosis threshold; the highest value observed in the fracture group was 79.3 mg/cm^3^. The fracture group was therefore contained entirely within the QCT-defined osteoporosis group, whereas 144 of the 461 patients without fracture (31.2%) also met the QCT criterion. Radiographic fracture assessment was available for all 800 patients, so this observation is not attributable to incomplete case ascertainment. The two endpoints are consequently not independent of one another, and the diagnostic estimates for fracture status must be interpreted in the light of this relationship.

**Figure 2:**
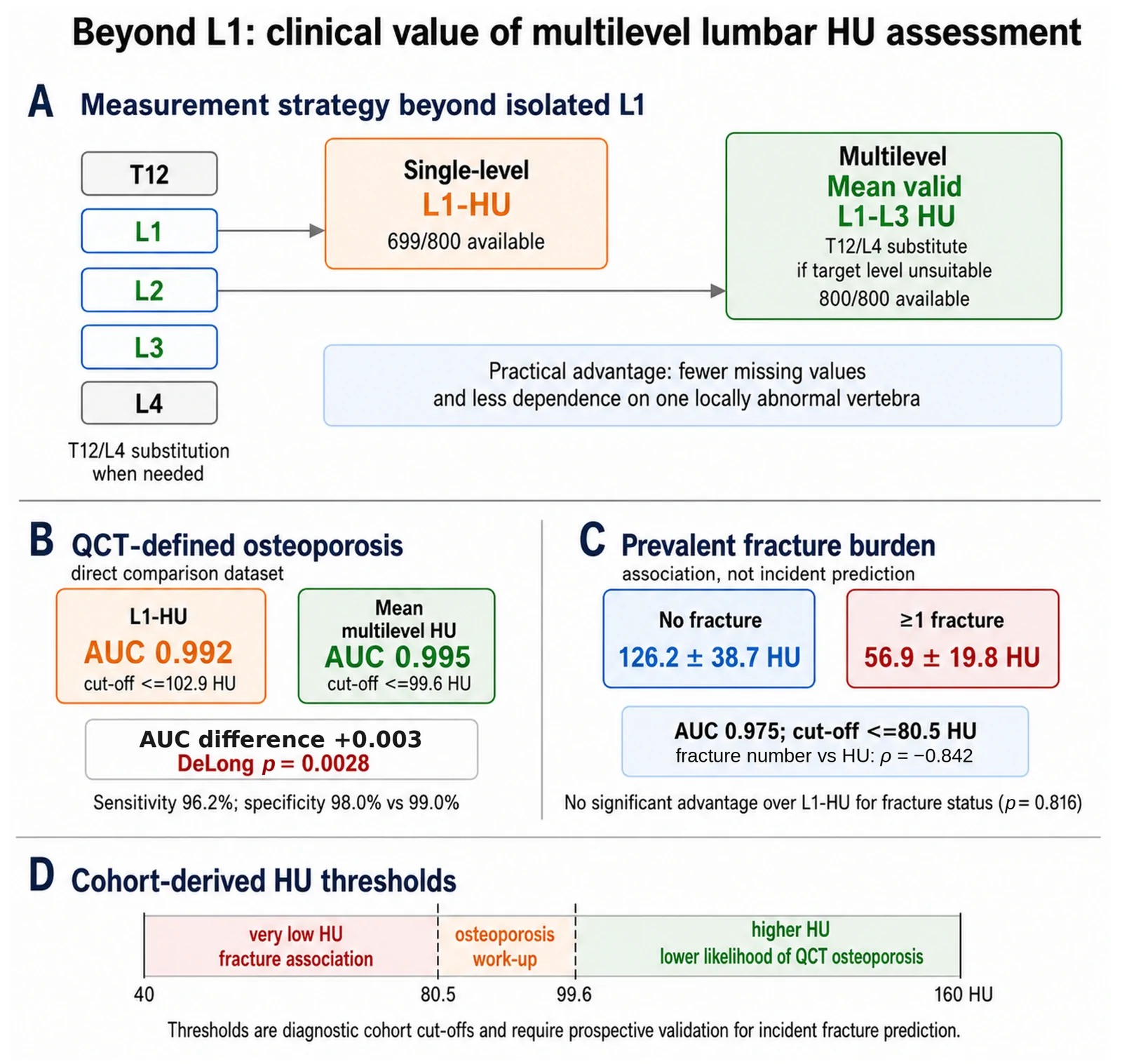
Multilevel lumbar HU assessment beyond isolated L1 measurement. (**A**) Schematic workflow comparing isolated L1-HU with the mean valid multilevel HU value across L1–L3, with predefined adjacent-level substitution when a target vertebra was fractured or technically unsuitable. (**B**) Direct comparison for QCT-defined osteoporosis in patients with available L1 measurements showed a small difference in the area under the curve in favor of mean multilevel HU. (**C**) Lower HU values were strongly associated with prevalent fracture status and fracture burden. (**D**) Internally derived, cohort- and protocol-specific HU thresholds for QCT-defined osteoporosis and prevalent fragility fracture status. The thresholds should not be interpreted as prospective incident fracture thresholds.

The diagnostic and practical value of the multilevel HU approach beyond isolated L1 measurement is summarized in Figure 2.

## 4. Discussion

### 4.1. Principal Findings

This study demonstrates that CT-based trabecular attenuation measurements of the lumbar spine are strongly associated with QCT-derived volumetric bone mineral density and prevalent fragility fracture status. The principal finding is that a multilevel HU approach using all valid available measurement levels provides a more widely obtainable measurement beyond isolated L1 assessment. A valid measurement was obtainable in all 800 patients using the multilevel approach compared with 699 of 800 (87.4%) using L1 alone. All patients with a prevalent fracture had a vBMD below the osteoporosis threshold, so the fracture endpoint is structurally dependent on the densitometric endpoint and the corresponding estimates must be interpreted accordingly. The mean multilevel HU value showed the strongest correlation with QCT-derived vBMD, and in direct comparison it was statistically superior to isolated L1-HU for QCT-defined osteoporosis, although the absolute difference in the area under the curve was only 0.003. For prevalent fragility fracture status the two parameters performed equivalently. These relationships and their clinical interpretation are summarized in Figure 2.

The internally derived threshold associated with QCT-defined osteoporosis in this cohort was approximately 100 HU, which is consistent with published clinical positions indicating that very low L1 attenuation values suggest a high likelihood of osteoporosis [31]. The present study extends this concept by showing that measurement availability can be improved when lumbar bone assessment is not restricted to L1 alone.

The title of the present study deliberately emphasizes bone assessment beyond L1. This is clinically relevant because L1 may be unavailable or unsuitable due to prevalent fracture, focal sclerosis, hemangioma, cement augmentation, metal artifacts, degenerative endplate changes, or other local confounders. In the present cohort, L1-HU was available in 699 of 800 patients, whereas the multilevel HU mean could be calculated in all 800 patients, and adjacent substitute levels were required four times more often in patients with a prevalent fracture (40.4%) than in those without (9.1%). Therefore, the advantage of multilevel assessment is not limited to a small statistical improvement in AUC. It also reflects greater practical applicability in a real-world clinical cohort with degenerative changes and prevalent fragility fractures.

### 4.2. Relationship Between HU and QCT-Derived vBMD

An important consideration in interpreting these results concerns the relationship between the two measurements compared. HU values and QCT-derived vBMD were obtained from the same CT acquisition, in the same vertebral bodies, and with reference to the same calibration phantom, and both are derived from the X-ray attenuation of the same trabecular bone. The very high correlation observed here must therefore be interpreted as a strong association between two related measurements of the same physical quantity, and not as validation against a methodologically independent reference standard. It should also be noted that correlation and regression statistics quantify association and do not by themselves establish agreement between methods; no formal agreement analysis was performed, so the extent to which HU values could substitute numerically for vBMD in an individual patient cannot be inferred from these data.

Consistent with this interpretation, the relationship between the two was close to a fixed proportionality, with a residual standard deviation of 6.9 HU and a coefficient of variation in the individual HU-to-vBMD ratio of 8.9%. The residual variation reflects differences in the sampled volume, the reconstruction plane, the operator and the analysis software rather than an independent biological comparison. The attenuation measurements were performed on pseudonymized image data without access to the QCT-derived vBMD values, so the agreement observed here cannot be attributed to knowledge of the comparator during region-of-interest placement; it reflects the shared physical basis of the two measurements. The generalized estimating equation gave a slope of 0.772 mg/cm^3^ per Hounsfield unit, which is the inverse of this proportionality and is consistent with the fixed relationship between attenuation and mineral density under a constant acquisition protocol. Validation of HU-based assessment against a genuinely independent standard, in particular incident fracture in a prospective cohort, therefore remains necessary.

### 4.3. Interpretation in the Context of Previous Literature

Previous biomechanical and clinical studies have shown that vertebral HU values correlate with bone mineral density, vertebral strength, and osteoporotic fracture status [20,21,22,23,24,25,26,27,28,29,30]. The present study confirms these observations in a large clinical cohort using QCT-derived vBMD as the reference standard. The close association between mean multilevel HU and QCT-vBMD indicates that cancellous attenuation provides a usable surrogate for trabecular bone density when measurements are performed in a standardized manner, subject to the methodological considerations set out in Section 4.2.

Large-scale opportunistic CT studies have frequently focused on L1 because this level is commonly included in thoracic and abdominal CT examinations and is technically easy to measure [22,26]. The present findings do not contradict the clinical usefulness of L1. Instead, they show that L1 is not always available and may not always be the most robust single representation of lumbar cancellous bone quality. This is particularly relevant in older cohorts, where prevalent vertebral fractures, degenerative endplate alterations, focal sclerosis, or prior interventions are common.

The present threshold of approximately 100 HU for QCT-defined osteoporosis aligns with prior clinical positions and large opportunistic CT literature [31]. At the same time, recent studies continue to show variability in proposed HU cut-offs for classifying bone as normal, osteopenic, or osteoporotic [38]. Differences in scanner type, tube voltage, reconstruction kernel, contrast phase, vertebral level, ROI size, and ROI placement may influence measured attenuation values. A recent systematic review and meta-analysis further supports the diagnostic value of lumbar CT HU thresholds for osteoporosis and osteopenia, but also reinforces that thresholds must be interpreted in the context of protocol and population [42]. Consistent with this, the fracture-associated threshold in the present cohort differed substantially between patients under and over 65 years of age (≤93.5 versus ≤80.5 HU), which further underlines that such cut-offs are population-dependent.

The importance of ROI methodology is particularly relevant for clinical implementation. Sobecki et al. emphasized that ROI size and placement affect clinical opportunistic CT trabecular HU measurement [39]. The present study used standardized ellipsoid cancellous ROI placement with reference-body adjustment, which likely contributed to the high correlation with QCT-vBMD. In this context, the multilevel approach should not be interpreted merely as averaging more measurements. Rather, it reduces the influence of local vertebral confounders and may provide a more representative regional estimate of lumbar trabecular bone quality.

Additional CT-based research supports the broader validity of cancellous bone density assessment. Recent CT versus micro-CT work across cervical, thoracic, and lumbar vertebrae further supports the structural relevance of CT-derived cancellous bone measurements [43]. These data are consistent with the present finding that HU values reflect a biologically meaningful trabecular bone parameter rather than a purely scanner-derived technical value.

### 4.4. Clinical Implications of Multilevel HU Assessment Beyond L1

The most direct clinical implication is that CT-based osteoporosis assessment should not be restricted to isolated L1 measurement when multiple valid lumbar levels are available. L1 remains useful when technically valid and when it is the only available vertebral level. However, when several valid lumbar or predefined adjacent substitute levels can be assessed, the mean multilevel HU value offers higher availability and a stronger association with QCT-vBMD than isolated L1-HU, with a statistically significant but numerically very small difference in discrimination.

**The magnitude of the diagnostic gain.** The increase in the area under the curve from 0.992 to 0.995 was statistically significant but should not be presented as a clinically important improvement in accuracy. The underlying data structure explains why the gain is necessarily small: trabecular attenuation is highly consistent across adjacent lumbar levels within an individual, with a mean within-patient standard deviation of 4.0 HU across L1–L3, a median absolute L1-to-L3 difference of 3.5 HU, and an intraclass correlation coefficient of 0.99. When adjacent levels agree this closely, averaging across three of them can reduce measurement noise only marginally. The clinically relevant advantages of the multilevel approach therefore lie in the 12.6 percentage-point gain in measurement availability and in reduced vulnerability to local vertebral confounders, not in superior discrimination. This gain is likely to be a conservative estimate. Patients with previous spinal surgery or pronounced scoliosis were not eligible and none was encountered, so surgical hardware, cement and spinal deformity of non-osteoporotic origin did not contribute to the loss of measurable levels; the need for substitution was attributable to osteoporotic vertebral compression deformity alone, which is reflected in the fourfold difference between patients with and without a prevalent fracture (40.4% versus 9.1%). Even under these conditions an adjacent substitute level was required in 22.4% of patients overall, and the proportion would be expected to be higher in an unselected population in which post-surgical and deformed spines are also present.

This is relevant for radiology, orthopedic, trauma, and spine-surgery workflows. A mean multilevel HU value can be derived from CT examinations that were obtained for other indications and can identify patients who may benefit from further osteoporosis evaluation, laboratory assessment, pharmacologic treatment consideration, fall-risk assessment, or fracture-prevention strategies. Because no additional radiation exposure is required, opportunistic HU assessment may help close the diagnostic gap in patients who undergo CT but do not receive formal bone density testing.

The clinical relevance of vertebral HU values also extends beyond diagnostic screening. Low HU values have been associated with mechanical complications in adult spinal deformity surgery and with implant-related outcomes such as screw loosening [44,45]. Other studies have linked vertebral HU values to the severity of osteoporotic thoracolumbar compression fractures [46]. Although the present study was not designed to evaluate surgical outcomes, these findings emphasize that CT-based bone quality assessment may have practical implications for preoperative risk stratification and treatment planning.

The fracture-status analysis requires cautious interpretation. Mean multilevel HU showed excellent discrimination between patients with and without prevalent fractures, and lower HU values were strongly associated with higher fracture burden. Nevertheless, this study was retrospective and cross-sectional. Therefore, the results should be interpreted as evidence of a strong association with prevalent skeletal fragility, not as proof of prospective fracture prediction. Moreover, every patient with a prevalent fracture had a vBMD below the osteoporosis threshold, so that the fracture group was entirely contained within the QCT-defined osteoporosis group. The apparent diagnostic separation for fracture status is therefore in large part a restatement of the separation at the vBMD threshold and does not constitute an independent test against a clinical event. Future longitudinal studies are required to determine whether multilevel lumbar HU parameters predict incident vertebral or sacral fractures independently of QCT-derived vBMD, age, sex, and established clinical risk factors.

### 4.5. Strengths and Limitations

*This study has several strengths:*

- It included a large clinical cohort of 800 patients and 2400 vertebral measurement levels across a wide range of ages and BMI values.
- HU measurements were directly compared with QCT-derived trabecular vBMD, which provides a volumetric reference less affected by degenerative extraskeletal changes than lumbar DXA [17,18].
- The study evaluated both densitometric classification and prevalent fragility fracture status, thereby linking the imaging biomarker to clinically relevant skeletal disease.
- The analysis directly compared single-level and multilevel HU strategies and used pairwise DeLong testing for correlated ROC curves.
- Another strength is the clinical realism of the cohort. The presence of unavailable or unsuitable L1 measurements, prevalent vertebral fractures, and degenerative changes reflects the population in which CT-based bone assessment is most likely to be useful. The fact that mean multilevel HU was available in all patients, whereas L1-HU was not, supports the practical rationale for a multilevel strategy.

*The study also has limitations:*

- First, HU and QCT-derived vBMD were not independent measurements, as set out in Section 4.2; the association between them is an agreement analysis and not external validation.
- Second, the study was conducted at a single center using one scanner and one acquisition protocol, and the cohort consisted of patients referred for dedicated bone density evaluation and examined with a calibration phantom. It therefore represents an enriched diagnostic referral population rather than a general screening population, which contributed to the high prevalence of QCT-defined osteoporosis and of prevalent fractures and is likely to have increased the apparent diagnostic separation. External validation in independent centers is required.
- Third, the retrospective, cross-sectional design precludes causal inference and does not permit assessment of incident fracture prediction; the reported HU thresholds are therefore diagnostic cut-offs for prevalent status and must not be read as prospective risk thresholds.
- Fourth, all patients with a prevalent fracture were within the QCT-defined osteoporotic range, so the two endpoints are structurally related and the fracture-status estimates are correspondingly optimistic.
- Fifth, all attenuation measurements were performed by a single reader and were not repeated, so neither inter-rater nor intra-rater agreement could be quantified in this cohort. The measurements were, however, performed on pseudonymized data without access to the QCT-derived vBMD values, and the two investigators were blinded to each other’s results, so the fracture endpoint was assessed independently of the attenuation measurement. The reproducibility of the method is therefore not demonstrated by the present data; published agreement data for the technique used here are available from our previous work on interobserver variability of HU measurement in vertebral cancellous bone [34,35] and from the inter-rater reliability analysis of Ullrich et al. [30], but formal agreement analysis in the present cohort remains a limitation.
- Sixth, patients with known malignant disease, pronounced scoliosis or previous spinal surgery were not eligible, and no patient with spinal surgery or pronounced scoliosis was encountered. This strengthens the internal validity of the fracture endpoint, since the fractures analyzed are fragility rather than pathological fractures, but it also means that the cohort was not affected by several of the confounders the multilevel approach is intended to overcome, so the availability advantage observed here is likely to be conservative.
- Seventh, adjacent-level substitution was required in 22.4% of patients; although a complete-case analysis reproduced the main findings, this introduces heterogeneity that warrants external validation.
- Eighth, CT acquisition parameters, reconstruction settings and scanner calibration affect HU values, and although reference-body adjustment was applied, the thresholds derived here are specific to a single scanner and protocol. In addition, the region of interest was sized individually according to vertebral body dimensions rather than held constant, which reflects clinical practice but introduces a further source of variability, since region-of-interest size is known to influence measured attenuation [39].
- Finally, the stratified and adjusted analyses were exploratory, were not corrected for multiple comparisons, and produced degenerate estimates in the smallest strata.

### 4.6. Future Directions

Future work should focus on prospective validation, scanner- and protocol-specific calibration, automated vertebral segmentation, and integration into structured radiology reporting systems. A clinically useful CT workflow should identify technically valid vertebral levels, exclude locally abnormal vertebral bodies, calculate a robust multilevel HU parameter, and generate an actionable recommendation for further osteoporosis evaluation when appropriate. A prospective study in a genuinely opportunistic setting, using routine CT examinations acquired for unrelated indications and without a calibration phantom, is the necessary next step before the present thresholds can be transferred to screening practice.

Recent developments in automated and deep learning-based opportunistic CT osteoporosis screening show that large-scale extraction of vertebral trabecular attenuation values is technically feasible and may support standardized population-level screening approaches [47]. In this context, the multilevel HU concept evaluated in the present study may provide a clinically interpretable and methodologically robust target parameter for automated CT-based bone assessment beyond isolated L1 measurement.

Recent expert consensus from the Asia-Pacific region further supports the role of artificial intelligence as an adjunctive tool for opportunistic osteoporosis screening and management, while emphasizing that AI-based approaches require transparent performance reporting, independent external validation, workflow integration, and ethical and regulatory oversight before broad clinical deployment [48]. In this framework, the multilevel lumbar HU parameter evaluated in the present study may serve as a clinically interpretable target for future automated CT-based bone assessment systems, rather than as a replacement for established densitometric and clinical risk assessment pathways.

Additional recent evidence further supports this implementation direction. Hiyama et al. evaluated average L1-L4 HU values and machine-learning models for osteoporosis prediction in spine-surgery candidates, with clinically relevant HU risk ranges generally converging around 90–130 HU [49]. In a separate spine-surgery cohort, CT-derived HU showed only moderate concordance with DXA and approximately 28% discordance, supporting the interpretation of HU as an opportunistic triage marker rather than a stand-alone replacement for densitometry [50]. Disease-specific data from patients with fibrosing interstitial lung disease also showed that mean T12-L2 HU derived from routine chest CT provided the highest Youden index and the most balanced sensitivity-specificity profile for osteoporosis screening [51]. A recent scoping review of AI-based CT osteoporosis assessment included 51 studies and found high diagnostic and screening performance, but also emphasized the predominance of retrospective single-center studies and the need for external validation [52].

## 5. Conclusions

Multilevel lumbar HU assessment provides a practical marker of trabecular bone quality beyond isolated L1 measurement. Its principal advantage is measurement availability: a valid value was obtainable in all 800 patients using the multilevel approach compared with 87.4% using L1 alone, and the approach is structurally less vulnerable to local vertebral confounders such as prevalent fracture, focal sclerosis and degenerative endplate change. The accompanying improvement in discrimination for QCT-defined osteoporosis was statistically significant but small in absolute terms (area under the curve 0.992 to 0.995) and should not be regarded as a clinically important gain in diagnostic accuracy; for prevalent fragility fracture status, no advantage over L1 was observed.

These findings support a pragmatic workflow in which L1 measurement remains appropriate where it is technically valid, while multilevel assessment should be preferred when several valid lumbar or predefined adjacent substitute levels are available. A threshold of approximately 100 HU was associated with QCT-defined osteoporosis in this cohort, and a lower threshold of approximately 80 HU with prevalent fragility fracture status. Both values were associated with the respective outcomes in the cohort and CT protocol studied and are internally derived cut-offs obtained in a diagnostic referral population examined with a calibration phantom. They are not established thresholds for general opportunistic screening, for clinical diagnosis, or for fracture prediction, and they should not be applied as such. External validation in independent cohorts and in a genuinely opportunistic setting is required before any clinical use, and prospective validation before any use for incident fracture prediction.

## Figures and Tables

**Table 1:** Demographic characteristics of the study population, overall and stratified by sex and by prevalent fragility fracture status. BMI, body mass index; Q1–Q3, first to third quartile. Women and men differed significantly in age and BMI, and patients with and without prevalent fracture differed significantly in age, BMI and sex distribution (all *p* < 0.001; Mann–Whitney U test for continuous and chi-square test for categorical variables).

Characteristic	Total (*n* = 800)	Women (*n* = 659)	Men (*n* = 141)	No fracture (*n* = 461)	Fracture (*n* = 339)
Age, years, mean ± SD	65.6 ± 13.6	66.6 ± 13.2	60.7 ± 14.1	60.0 ± 12.8	73.2 ± 10.7
Age, years, median (Q1–Q3)	67 (57–76)	67 (58–78)	59 (52–74)	60 (52–69)	76 (65–81)
Age, years, range	24–92	24–92	33–85	24–85	37–92
Age ≥ 65 years, *n* (%)	448 (56.0)	391 (59.3)	57 (40.4)	193 (41.9)	255 (75.2)
BMI, kg/m^2^, mean ± SD	26.7 ± 6.6	26.3 ± 6.7	28.6 ± 5.6	28.0 ± 7.5	25.0 ± 4.5
BMI, kg/m^2^, median (Q1–Q3)	25.3 (23.0–29.4)	25.1 (22.5–28.7)	27.0 (24.5–33.1)	25.5 (23.5–31.6)	24.6 (20.9–27.6)
BMI ≥ 30 kg/m^2^, *n* (%)	176 (22.0)	116 (17.6)	60 (42.6)	129 (28.0)	47 (13.9)

**Table 3:** Correlations between mean QCT-vBMD, mean multilevel HU, age and fracture burden.

Analysis	*n*	Pearson *r*	Spearman *ρ*	*p*-Value
Mean QCT-vBMD vs. mean multilevel HU	800	0.989	0.988	<0.001
Mean QCT-vBMD vs. age	800	−0.708	−0.676	<0.001
Mean multilevel HU vs. age	800	−0.705	−0.672	<0.001
Fracture number vs. mean multilevel HU	800	—	−0.842	<0.001
Fracture number vs. mean QCT-vBMD	800	—	−0.839	<0.001
Fracture number vs. age	800	—	0.495	<0.001

**Table 4:** Diagnostic performance of single-level and mean multilevel HU for both endpoints in the full cohort of 800 patients, each parameter evaluated in the patients in whom it was obtainable. Areas under the curve are given with DeLong confidence intervals and sensitivity and specificity with Wilson score intervals; cut-off values are internally derived Youden-index thresholds.

Parameter	*n*	AUC (95% CI)	Cut-Off	Sensitivity, % (95% CI)	Specificity, % (95% CI)
**Endpoint: QCT-defined osteoporosis (vBMD < 80 mg/cm^3^)**					
L1-HU	699	0.992 (0.988–0.996)	≤102.9	96.2 (93.9–97.7)	98.0 (95.7–99.1)
L2-HU	738	0.993 (0.990–0.997)	≤95.2	93.7 (91.0–95.6)	100 (98.8–100)
L3-HU	750	0.996 (0.994–0.998)	≤96.1	97.3 (95.3–98.4)	97.1 (94.6–98.5)
**Mean multilevel HU**	800	0.996 (0.994–0.998)	≤99.6	96.3 (94.2–97.6)	99.1 (97.3–99.7)
**Endpoint: prevalent fragility fracture status**					
L1-HU	699	0.971 (0.960–0.982)	≤81.3	89.1 (84.6–92.3)	93.9 (91.3–95.8)
L2-HU	738	0.970 (0.960–0.981)	≤79.4	89.8 (85.7–92.8)	91.9 (89.0–94.0)
L3-HU	750	0.970 (0.961–0.980)	≤81.4	92.3 (88.7–94.7)	91.4 (88.4–93.6)
**Mean multilevel HU**	800	0.975 (0.966–0.984)	≤80.5	91.4 (88.0–94.0)	93.5 (90.9–95.4)

**Table 5:** Fragility fracture burden per patient. Counts include vertebral body fractures and sacral fracture sites; a bilateral sacral insufficiency fracture is counted as two sites. Table 5 and Table 6 report vertebral body fractures only and therefore give a lower total.

Parameter	Result
Total number of patients	800
No fracture	461/800 (57.6%)
At least one fracture	339/800 (42.4%)
One fracture	71/800 (8.9%)
Two fractures	66/800 (8.2%)
Three fractures	88/800 (11.0%)
Four fractures	37/800 (4.6%)
Five fractures	45/800 (5.6%)
Six fractures	19/800 (2.4%)
Seven fractures	13/800 (1.6%)

**Table 6:** Topographic fracture patterns per patient.

Fracture Pattern Per Patient	*n*
Thoracic spine only	51
Lumbar spine only	53
Thoracic + lumbar spine	119
Thoracic spine + sacrum	47
Lumbar spine + sacrum	22
Thoracic + lumbar spine + sacrum	41
Sacrum only	6

**Table 7:** Regional distribution, most frequent locations and Genant severity grade of the 834 vertebral body fractures.

Vertebral Body Fractures	*n*	Proportion, %
**Region**		
Thoracic spine	470	56.4
Lumbar spine	364	43.6
**Most frequent locations**		
L1	135	16.2
T7	104	12.5
T12	98	11.8
T8	88	10.6
L2	86	10.3
L3	72	8.6
L4	60	7.2
**Genant severity grade**		
Grade 1	400	48.0
Grade 2	365	43.8
Grade 3	69	8.3

**Table 8:** Direct pairwise comparison of L1-HU and mean multilevel HU, including the complete-case analysis restricted to patients with valid measurements at all of L1, L2 and L3.

Analysis Set	Endpoint	Parameter	AUC	Cut-Off	DeLong *p*
Valid L1-HU (*n* = 699)	QCT osteoporosis	L1-HU	0.992	≤102.9 HU	Reference
Valid L1-HU (*n* = 699)	QCT osteoporosis	Mean multilevel HU	0.995	≤99.6 HU	0.0028
Valid L1-HU (*n* = 699)	Fragility fracture	L1-HU	0.971	≤81.3 HU	Reference
Valid L1-HU (*n* = 699)	Fragility fracture	Mean multilevel HU	0.971	≤80.2 HU	0.816
**Complete L1–L3 (*n* = 610)**	QCT osteoporosis	L1-HU	0.992	≤101.8 HU	Reference
**Complete L1–L3 (*n* = 610)**	QCT osteoporosis	Mean multilevel HU	0.995	≤99.1 HU	0.0005
**Complete L1–L3 (*n* = 610)**	Fragility fracture	L1-HU	0.968	≤81.3 HU	Reference
**Complete L1–L3 (*n* = 610)**	Fragility fracture	Mean multilevel HU	0.968	≤80.2 HU	0.96

**Table 9:** Diagnostic performance of mean multilevel HU within prespecified strata. Analyses were exploratory and were not corrected for multiple comparisons. Areas under the curve of 1.000 result from complete separation of the two groups in relatively small subgroups and do not indicate perfect diagnostic accuracy.

Subgroup	*n*	AUC, QCT Osteoporosis (95% CI)	AUC, Fragility Fracture (95% CI)	Fracture Cut-Off
Women	659	0.996 (0.993–0.999)	0.970 (0.959–0.981)	≤80.5 HU
Men	141	1.000 (separation)	1.000 (separation)	≤82.8 HU
Age < 65 years	352	1.000 (separation)	0.982 (0.971–0.993)	≤93.5 HU
Age ≥ 65 years	448	0.991 (0.984–0.997)	0.971 (0.957–0.985)	≤80.5 HU
BMI < 30 kg/m^2^	624	0.994 (0.991–0.998)	0.969 (0.957–0.980)	≤80.5 HU
BMI ≥ 30 kg/m^2^	176	1.000 (separation)	0.996 (0.991–1.000)	≤93.5 HU

**Table 10:** Multivariable logistic regression for prevalent fragility fracture status (800 patients, 339 events).

Predictor	Odds Ratio	95% CI	*p*-Value
Mean multilevel HU, per 10 HU lower	5.98	4.26–8.38	<0.001
Age, per 10 years older	0.69	0.50–0.94	0.020
Male sex	1.40	0.56–3.53	0.47
BMI, per 5 kg/m^2^ higher	1.72	1.28–2.30	<0.001

## Data Availability

The data presented in this study are available from the corresponding author upon reasonable request, subject to institutional and ethical restrictions. The imaging data are held at the Institute for Diagnostic and Interventional Radiology/Neuroradiology, Westkuestenklinikum Heide, Germany.

## Abbreviations

The following abbreviations are used in this manuscript:

AUC	Area under the curve
Auto mA	Automatic tube current modulation
BMI	Body mass index
CI	Confidence interval
CT	Computed tomography
DXA	Dual-energy X-ray absorptiometry
GE	General Electric
GEE	Generalized estimating equations
HU	Hounsfield units
ICC	Intraclass correlation coefficient
kVp	Peak kilovoltage
L	Lumbar vertebral body level
MRI	Magnetic resonance imaging
*n*	Number
NPV	Negative predictive value
OR	Odds ratio
PPV	Positive predictive value
Q1-Q3	First and third quartile
QCT	Quantitative computed tomography
ROC	Receiver operating characteristic
ROI	Region of interest
SD	Standard deviation
STROBE	Strengthening the Reporting of Observational Studies in Epidemiology
T	Thoracic vertebral body level
vBMD	Volumetric bone mineral density
*p*	Probability value
*r*	Pearson correlation coefficient
*ρ*	Spearman rank correlation coefficient
